# CINC-2 and *miR-199a-5p* in EVs secreted by transplanted Thy1^+^ cells activate hepatocytic progenitor cell growth in rat liver regeneration

**DOI:** 10.1186/s13287-023-03346-z

**Published:** 2023-05-16

**Authors:** Norihisa Ichinohe, Naoki Tanimizu, Keisuke Ishigami, Yusuke Yoshioka, Naoki Fujitani, Takahiro Ochiya, Motoko Takahashi, Toshihiro Mitaka

**Affiliations:** 1 Department of Tissue Development and Regeneration, Research Institute for Frontier Medicine, Sapporo Medical University School of Medicine, South-1, West-17, Chuo-Ku, Sapporo, 060-8556 Japan; 2 Division of Regenerative Medicine, Center for Stem Cell Biology and Regenerative Medicine, The Institute of Medical Science, University of Tokyo, Tokyo, Japan; 3 Division of Molecular and Cellular Medicine, National Cancer Center Research Institute, Tokyo, Japan; 4 Department of Molecular and Cellular Medicine, Institute of Medical Science, Tokyo Medical University, Tokyo, Japan; 5 Department of Biochemistry, Sapporo Medical University School of Medicine, Sapporo, Japan

**Keywords:** Thy1^+^ mesenchymal cells, Extracellular vesicles, Hepatocytic progenitor cells, Small hepatocytes, miR-199a-5p, CINC-2, IL17RB signal

## Abstract

**Background:**

Small hepatocyte-like progenitor cells (SHPCs) are hepatocytic progenitor cells that transiently form clusters in rat livers treated with retrorsine (Ret) that underwent 70% partial hepatectomy (PH). We previously reported that transplantation of Thy1^+^ cells obtained from d-galactosamine-treated livers promotes SHPC expansion, thereby accelerating liver regeneration. Extracellular vesicles (EVs) secreted by Thy1^+^ cells induce sinusoidal endothelial cells (SECs) and Kupffer cells (KCs) to secrete IL17B and IL25, respectively, thereby activating SHPCs through IL17 receptor B (RB) signaling. This study aimed to identify the inducers of IL17RB signaling and growth factors for SHPC proliferation in EVs secreted by Thy1^+^ cells (Thy1-EVs).

**Methods:**

Thy1^+^ cells isolated from the livers of rats treated with d-galactosamine were cultured. Although some liver stem/progenitor cells (LSPCs) proliferated to form colonies, others remained as mesenchymal cells (MCs). Thy1-MCs or Thy1-LSPCs were transplanted into Ret/PH-treated livers to examine their effects on SHPCs. EVs were isolated from the conditioned medium (CM) of Thy1-MCs and Thy1-LSPCs. Small hepatocytes (SHs) isolated from adult rat livers were used to identify factors regulating cell growth in Thy1-EVs.

**Results:**

The size of SHPC clusters transplanted with Thy1-MCs was significantly larger than that of SHPC clusters transplanted with Thy1-LSPCs (*p* = 0.02). A comprehensive analysis of Thy1-MC-EVs revealed that *miR-199a-5p*, cytokine-induced neutrophil chemoattractant-2 (CINC-2), and monocyte chemotactic protein 1 (MCP-1) were candidates for promoting SHPC growth. Additionally, *miR-199a-5p* mimics promoted the growth of SHs (*p* = 0.02), whereas CINC-2 and MCP-1 did not. SECs treated with CINC-2 induced *Il17b* expression. KCs treated with Thy1-EVs induced the expression of CINC-2, *Il25*, and *miR-199a-5p*. CM derived from SECs treated with CINC-2 accelerated the growth of SHs (*p* = 0.03). Similarly, CM derived from KCs treated with Thy1-EVs and *miR-199a-5p* mimics accelerated the growth of SHs (*p* = 0.007). In addition, although *miR-199a*-overexpressing EVs could not enhance SHPC proliferation, transplantation of *miR-199a*-overexpressing Thy1-MCs could promote the expansion of SHPC clusters.

**Conclusion:**

Thy1-MC transplantation may accelerate liver regeneration owing to SHPC expansion, which is induced by CINC-2/IL17RB signaling and *miR-199a-5p* via SEC and KC activation.

**Graphical Abstract:**

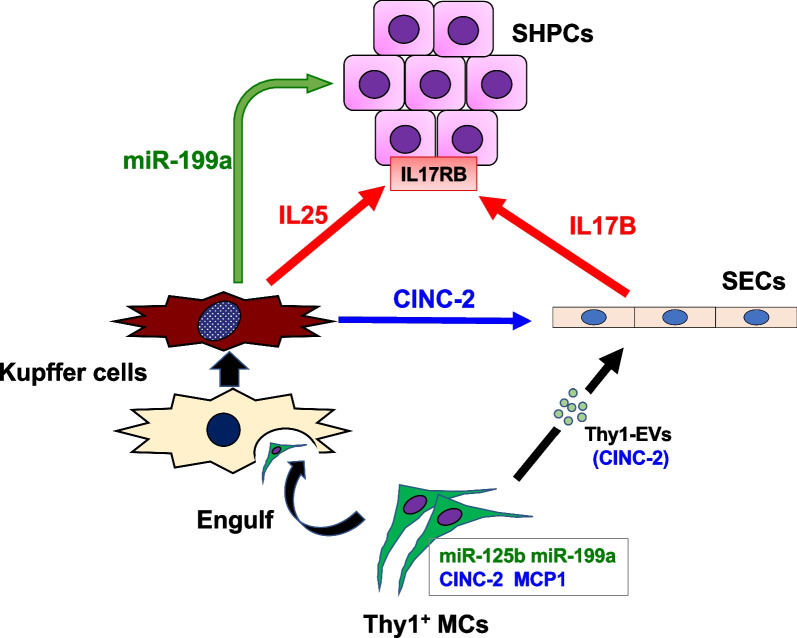

**Supplementary Information:**

The online version contains supplementary material available at 10.1186/s13287-023-03346-z.

## Background

Thy1 (CD90) was identified as a lymphocyte marker more than 50 years ago [1]; thereafter, it has been demonstrated that Thy1 is expressed on the surface of diverse cell types [2], particularly bone marrow-derived stem cells [3]. Oval cells are liver stem/progenitor cells (LSPCs) that emerge in the regenerating livers of rats treated with hepatotoxins, such as 2-acetylaminofluorene, d-galactosamine (GalN), 3-diethoxycarbonyl-1, 4-dihydrocollidine, and dipin, in combination with or without partial hepatectomy (PH) [4–7]. Petersen et al. [8] reported that oval cells express Thy1 on their cell membranes and proposed that these cells are derived from the bone marrow [9]. However, whether Thy1 is a specific marker of oval cells is controversial [10, 11].

Small hepatocytes (SHs) are a subpopulation of hepatocytes found in rats, mice, and humans. SHs isolated from healthy adult rat [12, 13], mouse [14], and human [15, 16] livers can clonally proliferate to form colonies and differentiate into mature hepatocytes (MHs) in vitro. We previously identified CD44 as a marker of SHs [17]. In GalN-induced rat liver injury, oval cells were observed in the periportal area, which differentiated into MHs by forming basophilic small-sized cells [18, 19]. Furthermore, we observed Thy1^+^ cells in the periportal area 2 days after GalN treatment. Immediately after the emergence of Thy1^+^ cells, CD44^+^ cells transiently appeared near the periportal area between Thy1^+^ cells and resident hepatocytes. CD44^+^ cells were then found to differentiate into MHs and lose CD44 expression [17, 20, 21]. The sequential histological alterations in GalN-treated livers were verified using sorted cells. When Thy1^+^ cells isolated from the liver at 2 days after GalN treatment were plated on culture dishes, most cells showed proliferation and fibroblast-like features. Subsequently, epithelial cell colonies appeared and expanded. Although a few colonies appeared, the cells expressed CD44 and Thy1, and were morphologically similar to SHs; they were termed as hepatocytic progenitor cells (HPCs) [20, 21]. The number of Thy1^+^ cells that could form epithelial colonies increased in the Thy1^+^ cell population isolated from the livers 3 days after GalN treatment. Thy1^+^ cells comprised epithelial colonies that sequentially converted their phenotypes from Thy1^+^/CD44^−^ to Thy1^−^/CD44^+^ through Thy1^+^/CD44^+^ and then differentiated into MHs [20, 21]. The other Thy1^+^ cells can proliferate and maintain a fibroblast-like appearance. The fibroblastic cells expressed desmin in their cytoplasm and were reported to be hepatic myofibroblasts [10, 11]. We termed these fibroblastic cells as mesenchymal cells (MCs).

Retrorsine (Ret) severely inhibits the replication of MHs. Therefore, the administration of Ret before PH suppresses liver regeneration. In Ret/PH-treated rat livers, small hepatocyte-like progenitor cells (SHPCs) emerge and aid the recovery of lost tissue [22, 23]. Although SHPCs expand rapidly, they require approximately 1 month to recover the original liver volume [22]. The morphology of SHPCs is similar to that of MHs; however, their size is much smaller than that of MHs. They share some phenotypic characteristics with fetal hepatoblasts, oval cells, and MHs; however, their origins remain unclear [24–26].

We previously reported that the transplantation of Thy1^+^ cells isolated from GalN-treated livers increased the number and size of SHPC clusters in Ret/PH-treated rat livers [27]. A comprehensive analysis of gene expression revealed that the transplantation of Thy1^+^ cells upregulated the expression of interleukin 17 receptor B (IL17RB) in SHPCs. IL17B and IL25, which are ligands of IL17RB, were expressed by sinusoidal endothelial cells (SECs) and Kupffer cells (KCs), respectively. Furthermore, we demonstrated that SHPC growth was stimulated by the administration of the extracellular vesicles (EVs) secreted by cultured Thy1-MCs via IL17RB signaling. Nevertheless, it remains unclear how Thy1-EVs activate IL17RB signaling to induce SHPC growth in the local environment of recipient livers. In the current study, we demonstrated that *miR-199a-5p*, *miR-125b-5p*, cytokine-induced neutrophil chemoattractant-2 (CINC-2), and monocyte chemotactic protein 1 (MCP-1) are abundant in Thy1-EVs. CINC-2 promotes IL17B expression in SECs. Additionally, Thy1^+^ cells induce the secretion of EVs containing *miR-199a-5p* and CINC-2 in KCs to promote SHPC proliferation and IL17B production in SECs, respectively. Hence, the acceleration of liver regeneration by transplanting Thy1-MCs may result from SHPC expansion activated by CINC-2/IL17RB signaling and *miR-199a-5p*.

## Methods

### Animals

F344 rats are inbred strains, and it is well known that immunological rejection of donor cells rarely occurs in cell transplantation experiments. Female F344/DuCrlCrlj rats (8 weeks old; Charles River Japan, Yokohama, Japan) were used as recipients. Male F344/Nslc rats (8–10 weeks old; Sankyo Lab Service Corporation, Inc., Tokyo, Japan) were used for obtaining donor cells. The F344/DuCrlCrlj strain genetically lacks dipeptidyl peptidase IV (DPPIV), whereas the F344/Nslc strain is wild and exhibits DPPIV expression. To identify donor cells in the recipient livers, we used DPPIV-positive cells isolated from F344/Nslc rat livers as donor cells and F344/DuCrlCrlj rats as the recipient. DPPIV-positivity can be demonstrated immunohistochemically and enzymohistochemically [28, 29]. All animals received appropriate care, and the experimental protocol was approved by the Committee of Laboratory Animals (Approval No.: 17-032, 17-033, 17-034, 19-055, and 20-058). The study was in line with the guidelines stipulated by Sapporo Medical University. For GalN-induced liver injury, GalN (75 mg/100 g body weight dissolved in phosphate-buffered saline [PBS]; Acros, Geel, Belgium, http://www.acros.com) was intraperitoneally administered. For the transplantation experiment, female F344 rats were administered two intraperitoneal injections of Ret (30 mg/kg body weight; Sigma-Aldrich, Co., St. Louis, MO, www.sigma-aldrich.com) at an interval of 2 weeks [22, 29]. Two weeks after the second injection, 70% PH was performed. Sorted Thy1^+^ (5 × 10^5^) cells and isolated EVs obtained from cultured Thy1^+^ cells were administered to Ret/PH livers (DPPIV^−^ rat) through the spleen. Forty rats were randomly divided into control and seven target groups (five rats per group). Twenty-seven rats were employed for cell isolation experiments. Accordingly, 67 rats were used in this study. The humane endpoints were established and monitored in accordance with the guidelines stipulated by Sapporo Medical University. No rats exhibited any signs of the established endpoints in this study. For histological analyses, rats were euthanized by incising the inferior vena cava via PBS perfusion under anesthesia, and the livers were resected for histological analyses. For all treatment methods, rats were anesthetized using a mixture of O_2_/N_2_ (1:1) and isoflurane.

### Sorting and culture of cells isolated from the liver

The isolation and subculture methods have been described previously [21, 27, 28, 30, 31]. Briefly, liver cells were isolated from DPPIV^+^ rats using the two-step collagenase perfusion method [17], following which the cell suspension was centrifuged at 50×*g* for 1 min. The supernatant and precipitate were used for isolating Thy1^+^ cells and MHs, respectively. Thy1^+^ cells were isolated from injured livers 3 days after GalN treatment (GalN-D3) [27]. SHs, MHs, SECs, and KCs were isolated from the liver of a healthy rat (8–12 weeks old). The cells were cultured as previously described [21] and then sorted via magnetic cell sorting. Mouse anti-rat Thy1 (Serotec, Raleigh, NC), mouse anti-rat SE-1 (Immuno-Biological Lab., Takasaki, Japan), and mouse anti-rat CD68 (Serotec) antibodies were used as primary antibodies for sorting Thy1^+^ cells, SECs, and KCs, respectively (Additional file 2: Table S1). SHs were isolated from a healthy liver as previously described [27]. After enumerating viable cells using the trypan blue exclusion test, cells were plated on each culture dish at a density of 1 × 10^5^ cells/mL. Thy1^+^ cells were plated on 10-cm culture dishes coated with rat tail collagen. SECs and KCs were plated on a 24-well plate coated with rat tail collagen. Furthermore, SHs were plated on hyaluronic acid (Sigma-Aldrich Co., St. Louis, MO)-coated dishes (1 mg/35-mm dish). SHs were cultured for 10 days and then subcultured on Matrigel-coated 12-well plates as previously described [31, 32]. The cells were cultured in DMEM/F12 medium (Sigma-Aldrich) supplemented with 20 mM HEPES (Dojindo Chemical Laboratories, Kumamoto, Japan), 25 mM NaHCO_3_ (Kanto Chemical Co. Inc., Tokyo, Japan), 30 mg/L l-proline (Sigma-Aldrich), 0.1% bovine serum albumin (Serologicals Proteins Inc., Kankakee, IL), 10 mM nicotinamide (Sigma-Aldrich), 1 mM ascorbic acid 2-phosphate (Fujifilm Wako Pure Chem., Osaka, Japan), 10 ng/mL epidermal growth factor (BD Biosciences, Bedford, MA), ITS-X (BD Biosciences), 10^−7^ M dexamethasone, and antibiotics. The medium was replenished every alternate day.

### Separation of Thy1-MCs

**Fig. 1:**
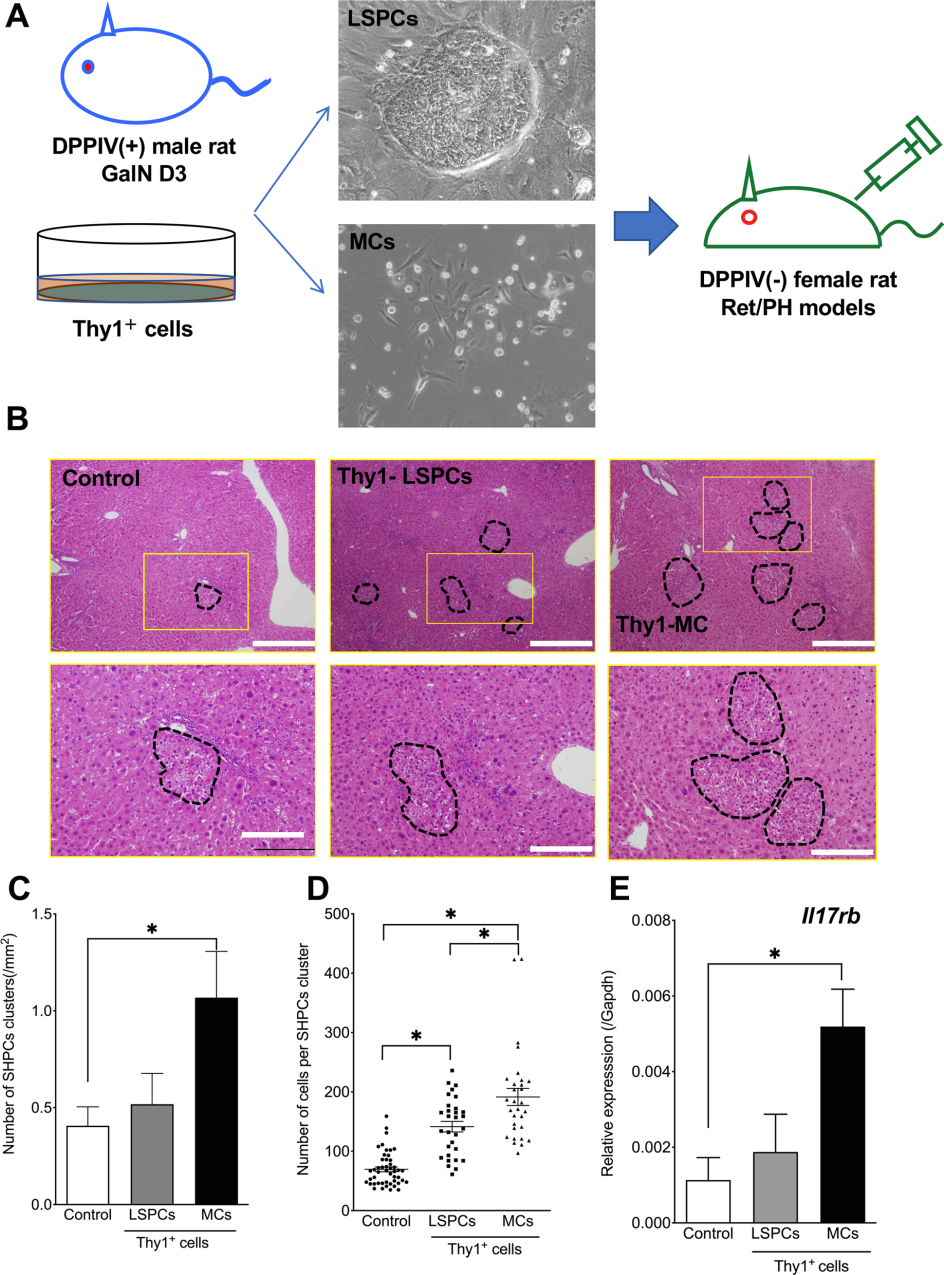
Transplantation of Thy1-LSPCs and Thy1-MCs to Ret/PH-treated rat livers. **A** Schematic diagram showing the cell transplantation method. **B** Photos of SHPC clusters (black dotted lines) in hematoxylin–eosin (HE)-stained livers transplanted with control (left), Thy1-LSPCs (center), and Thy1-MCs (right). Inset depicts enlarged typical SHPCs (yellow square) in livers with and without Thy1^+^ cell transplantation. Fine fat droplets (empty vesicles) are observed in the cytoplasm of SHPCs. Scale bar, 500 μm. **C** The number of SHPC clusters per area and **D** area of the cluster were measured 14 days after transplantation. **E** *Il17rb* expression in SHPCs of livers that underwent Thy1^+^ cell transplantation was verified via qRT–PCR. Asterisks indicate significant differences at *p* < 0.05

The separation methods have been described previously [20, 21]. Thy1^+^ cells obtained after GalN-D3 treatment were seeded into 10-cm culture dishes and cultured for 7 days. The proliferated cells comprised epithelial cells and MCs (Fig. 1A). The epithelial cells formed colonies resembling normal SHs and exhibited the morphological characteristics of LSPCs. These colonies were detached and collected from the dish using a cell dissociation buffer on day 7. The remaining MCs on the dish were detached using 2% trypsin/0.02% EDTA/PBS. Subsequently, viable cells were enumerated, and epithelial cells and MCs (5 × 10^5^ cells/mL) were transplanted into Ret/PH-treated rat livers through the spleen. For isolating EVs, separated epithelial cells and MCs (1 × 10^6^ cells) were seeded into 10-cm dishes and cultured in a serum-free medium for 2 days. EVs were isolated from the conditioned medium (CM).

### Flow cytometry

To characterize Thy1^+^ cells, flow cytometry was performed as previously reported [32]. Briefly, Thy1^+^ cells were cultured for 7 days, trypsinized, and centrifuged at 150×*g* for 5 min. After removing the supernatant, the pellets were washed with PBS and incubated with mouse anti-rat antibodies against CD90, CD73, and CD44 in DMEM containing 10% FBS for 30 min at 4 °C. The pellets were collected and centrifuged at 150×*g* for 5 min. After washing with PBS containing 2% FBS (wash buffer), the pellets were incubated with rabbit anti-mouse IgG (H + L) antibodies conjugated with Alexa Fluor 488 in DMEM containing 10% FBS for 30 min at 4 °C (Additional file 2: Table S1). The pellets were washed again after centrifugation. Subsequently, they were suspended in a wash buffer containing propidium iodide solution and were passed through a 35-µm cell strainer (Falcon, Corning Inc). The cells were analyzed using FACSCanto flow cytometer (BD Biosciences, San Jose, USA). All antibodies used in this study are listed in Additional file 2: Table S1. Data were analyzed using Kaluza Flow Cytometry Software version 1.1 (Beckman Coulter, Inc., Brea, USA).

### Immunohistochemistry

The antibodies used for immunohistochemistry are listed in Additional file 2: Table S1. Recipient rats were euthanized 14 days after transplantation and their livers were immediately harvested and sliced on ice. Five-mm thin sections were embedded in Tissue-Tek (Sakura Finetechnical Co., Tokyo, Japan), frozen in isopentane/liquid nitrogen, and stored at − 80 °C until use. Some sections were fixed in 10% paraformaldehyde/buffered PBS. Enzyme- and immuno-histochemistry for DPPIV were performed to identify donor cells [20, 28]. SHPCs in Ret/PH-treated rat livers were identified as clusters containing > 10 small-sized hepatocytes, and areas covered by SHPCs in the livers were measured using cellSens Dimension software (OLYMPUS Corp., Tokyo, Japan).

### Laser microdissection and gene expression analysis

SHPC clusters in recipient livers were obtained via laser microdissection, according to the manufacturer’s protocol [27, 32]. Total RNA was isolated from the collected cells using RNeasy Mini Kit (Qiagen, Hilden, Germany). Briefly, 7-μm-thick frozen sections were prepared from liver tissues and stained with hematoxylin. SHPC clusters were dissected under a microscope using an ultraviolet laser (MMI CellCut; Molecular Machines & Industries, Glattbrugg, Switzerland). Further, the gene expression patterns were analyzed using quantitative reverse transcription polymerase chain reaction (qRT–PCR).

### MicroRNA (miRNA) extraction, microarray analysis, and qRT–PCR

Microarray analysis of miRNAs has been previously described [32]. miRNAs were extracted from EVs using Qiazol lysis reagent and miRNeasy mini kit (Qiagen). A comprehensive analysis of miRNA expression was performed using 3D-Gene miRNA Labeling kit and 3D-Gene miRNA Oligo Chip (Toray Industries, Inc. Tokyo, Japan), which was designed to detect 727 miRNA sequences registered in miRBase release 20. For gene expression analysis, miRNAs were transcribed into cDNA using TaqMan MicroRNA Reverse Transcription kit (Applied Biosystems, Foster City, USA) and reverse transcription primers provided with TaqMan miRNA assay kit (Applied Biosystems). The cDNA products were analyzed using TaqMan miRNA sequence-specific probes for U6 small nuclear 2 (*U6*), *miR-125b-5p* (*miR-125b*), *miR-145-5p* (*miR-145*), *miR-199a-5p* (*miR-199a*), *miR-451-5p* (*miR-451*), *miR-3473-5p* (*miR-3473*), and *let-7f* Premix Ex Taq (Takara) and the ABI Prism 7500 sequence detection system (Applied Biosystems) [32]. The primers used in this study are listed in Additional file 2: Table S2. All miRNA microarray data are registered in the GEO database (Accession No. GSE222517).

### qRT–PCR

The details of qRT–PCR analysis have been previously reported [27, 32]. Briefly, RNA was reverse-transcribed using OmniScript RT Kit (Qiagen) and random hexamers as primers. qRT–PCR analyses were performed using TaqMan RNA sequence-specific probes and Premix Ex Taq (Takara). The reactions were performed in triplicate in 96-well optical plates for all samples using ABI Prism 7500 cycler (Applied Biosystems). The relative expression of each gene was normalized to that of the control *GAPDH*. The primers used in this study are listed in Additional file 2: Table S2.

### Morphological analyses of cultured cells

The details of morphological analyses have been previously reported [27, 32]. The cultured cells were photographed under a phase-contrast microscope equipped with a CCD camera (Olympus Corp., Tokyo, Japan) to enumerate the colonies and cells per colony. After randomly selecting ten fields per dish or well, at least three dishes or wells were analyzed per experiment and at least two independent experiments were performed. All acquired images were analyzed using cellSens Dimension software (OLYMPUS Corp.).

### Measurement of the labeling index (LI)

According to previous studies [27, 32], cultured cells were treated with 40 µM 5-bromo-2ʹ-deoxyuridine (BrdU) for 18 h before fixation. The cells were fixed with absolute cold ethanol for 15 min and incubated first with 2 N HCl for 30 min at room temperature (RT) and then with 0.6% hydrogen peroxide in absolute methanol for 30 min at RT. The cells were blocked with BlockAce for 30 min at RT and incubated with a mouse anti-BrdU antibody for 60 min. The cells were washed with PBS and subsequently incubated with a biotinylated anti-mouse antibody (Vector Laboratories, Burlingame, CA) for 30 min at RT. Then, the cells were incubated with an avidin–biotin complex solution (VECTASTAIN ABC kit; Vector Laboratories) and treated with 3,3-diaminobenzidine for color development. Cells with BrdU-positive nuclei were enumerated to determine LI.

### Isolation of EVs

EVs were separated from CM as previously described [32, 33]. Thy1^+^ cells were cultured for 5 days and washed with PBS, and CM was replaced with serum-free DMEM (Sigma-Aldrich Co.). After 48 h, CM was collected and centrifuged for 10 min at 2000×*g* and 4 °C. The supernatant was filtered through a 0.22-μm filter (Millipore, Billerica, USA) to remove cellular debris. To prepare EVs, CM was ultracentrifuged for 70 min at 110,000×*g* and 4 °C [32, 33]. The supernatant contained CM without EVs (CM-EVs), and the precipitate was resuspended in 200 µL of saline. The concentration of EVs was measured using NanoDrop 1000 spectrometer (Thermo Fisher Scientific, Inc, Waltham, MA), and protein concentrations were determined using BCA assay kit (Thermo Fisher Scientific, Inc.).

### Mass spectrometry (MS)

To characterize EVs, proteome analysis was performed using MS. Aliquots containing 15 μg of total protein extracted from EVs were reductively alkylated with 10 mM dithiothreitol (Fujifilm Wako, Japan) followed by treatment with 20 mM iodoacetamide (Fujifilm Wako). The reductively alkylated proteins were digested with 0.75 μg of sequence grade trypsin/Lys-C (Promega, WI, USA) for 16 h at 37 °C. The resultant peptides were desalted with a styrene divinylbenzene polymer tip column (GL Science, Tokyo, Japan). The desalted peptides were completely evaporated using a centrifugal evaporator. Finally, the obtained peptides were redissolved in 10 μL of ultrapure water containing 0.1% formic acid. This sample solution was subjected to MS using Orbitrap Q Exactive Plus (Thermo Fisher Scientific, Inc.) coupled to the EASY-nLC system equipped with a C18 column (0.075 mm × 125 mm, Nikkyo Technos, Tokyo, Japan). The sample was eluted using an acetonitrile gradient from 0 to 30% within 90 min at a flow rate of 300 nL/min. All data were obtained in a data-dependent mode, and tandem MS (MS/MS) was performed based on high energy collision-induced dissociation. The MS/MS data were processed using MaxQuant software 1.6.3.3 [34]. Peptide searches were performed by referring to the proteomic data of *Rattus norvegicus* obtained from UniProtKB (https://www.uniprot.org/). The proteomic data are registered in the Proteome Xchange Consortium database (Accession No: PXD039384).

### Administration of EVs to Ret/PH livers

Thy1^+^ cells were cultured for 7 days. Then, the cells and CM were used for transplantation and EV isolation, respectively. The number of EVs secreted by 5 × 10^5^ donor Thy1^+^ cells in 48 h was estimated. The pellet obtained after ultracentrifugation of CM was resuspended in 200 µL of saline and administered to recipient livers through the spleen using a low dead-space syringe with a 21-gauge needle (NIPRO, Tokyo, Japan) [32].

### Transfection of miRNA mimics into cultured SHs

SHs cultured on Matrigel-coated 12-well plates at a density of at 5 × 10^4^ cells/well were transfected with TaqMan miRNA mimics corresponding to *miR-125b-5p* (Applied Biosystems, MC), *miR-145-5p* (miR-145), *miR-199a-5p* (miR-199a), *miR-451-5p* (miR-451), *miR-3473-5p* (miR-3473), and *miR-let-7f* as well as negative controls (final concentration: 50 nM; Applied Biosystems, Cat No. 4464058). Transfection was performed using Lipofectamine RNAiMAX (Invitrogen, Carlsbad, USA) according to the manufacturer’s instructions [32]. After 2 days, the medium was replenished, cells were cultured for 5 days, and colony growth was evaluated.

### Analysis of cytokine content

Isolated EVs were lysed in RIPA buffer containing 1 mM protease inhibitors (Sigma-Aldrich). The resulting lysates were assayed to detect Thy1-MC-derived proteins using a custom-designed Quantibody rat-specific protein array (RayBiotech, Peachtree Corners, USA, cat. No. QAR-CAA-67) at Cosmo Bio, Ltd. (Tokyo, Japan) [32].

### Overexpression of miR-199a-5p in EVs derived from Thy1^+^ cells using lentivirus

Transfection was performed using XMIRXpress vector (SBI System Biosciences) according to the manufacturer’s instructions [32, 35]. Briefly, 293 TN (3 × 10^6^) cells were inoculated into 75-cm^2^ culture flasks. Then, 2 µg of transfer plasmid (*miR-125b-5p*, *miR-199a-5p*, or nontarget miRNA) and 20 µL of pPACKH1 plasmid were mixed with 800 µL of serum-free DMEM in tubes via pipetting. Next, 24 µL of PureFection reagent (SBI System Biosciences) was added to the tubes, vortexed vigorously, and incubated at RT for 15 min. The mixtures were added dropwise into the flask and swirled to disperse evenly. After 2 days, media were collected in 12-mL tubes and centrifuged at 3000×*g* for 15 min to remove cell debris. The viral supernatant was added to the culture medium. EVs produced via transfected MCs were collected and analyzed for the expression of *miR-125b-5p* and *miR-199a-5p* using qRT–PCR. The ability of EVs to induce SHPC growth in Ret/PH rat models was analyzed.

### Effects of miR-199a mimics as well as CM derived from SECs treated with CINC-2 and KCs treated with Thy1-EVs on SH growth

SECs and KCs isolated from a healthy rat liver were cultured on type I collagen-coated 12-well plates (Corning Inc). Three hours after seeding, the culture medium of SECs and KCs was replaced with a medium containing CINC-2 or Thy1-EVs and *miR-199a* mimics, respectively. These cells were cultured for 2 days, and CM was obtained. Next, 100 μL of CM was added to the SH culture medium, and SHs were then cultured for 7 days. CINC-2-treated SEC-CM was continuously added for 7 days. Contrarily, KC-CM treated with Thy1-EVs was added only for the initial 2 days. The size of SH colonies was measured 7 days after plating.

### Statistical analysis

Array data were analyzed using MultiExperiment Viewer software (https://mev.tm4.org/). Microarray data were analyzed using Student’s *t* test. All other data were compared using Tukey’s multiple comparison test. Statistical analyses were performed using GraphPad Prism software (GraphPad Software, La Jolla, CA). A *p *value of < 0.05 was considered to indicate statistical significance. The experimental results are expressed as mean ± standard error.

## Results

### Thy1-MCs accelerated SHPC growth through IL17RB signaling

Recipient livers were histologically examined 14 days after the transplantation of Thy1-positive cells. There were more SHPC clusters containing small cells in livers transplanted with Thy1-MCs than in those transplanted with Thy1-LSPCs and nontransplanted liver controls. SHPC clusters were randomly distributed in liver lobules and were not limited to a specific area (Fig. 1B). The number of clusters in livers transplanted with Thy1-MCs was approximately twofold higher than that in the control (Fig. 1C). The number of cells per cluster was significantly higher in Thy1-MCs than in Thy1-LSPCs and controls (Fig. 1D).

**Fig. 2:**
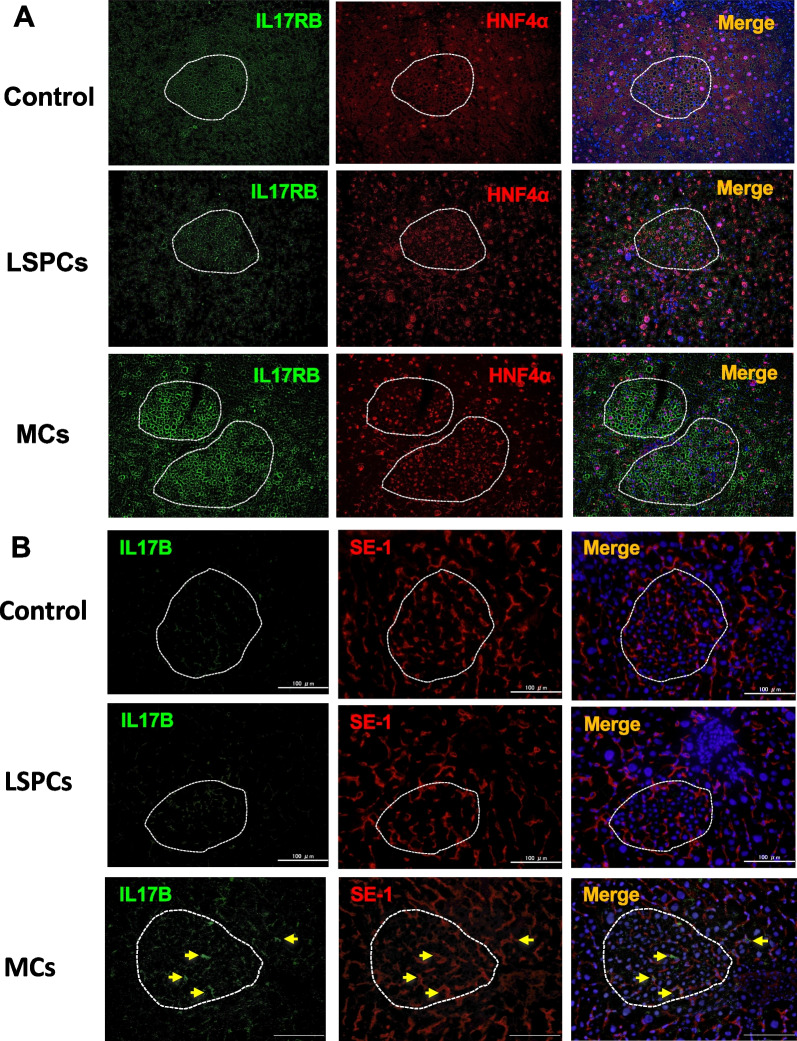
IL17RB and IL17B expression in SHPCs with and without Thy1^+^ cell transplantation. Double immunohistochemistry for IL17RB/HNF-4α (A) and IL17B/SE-1 (B) in Ret/PH-treated rat livers transplanted with control, Thy1-LSPCs, and Thy1-MCs. SHPC clusters are enclosed by dotted lines. Scale bar, 100 μm

Thy1-EVs have been reported to induce SHPC growth through IL17RB signaling in Ret/PH-treated rat livers [27]. To verify IL17RB expression in SHPCs, we first performed qRT–PCR and revealed that SHPCs derived from livers transplanted with Thy1-MCs showed significantly increased *Il17rb* expression compared with those derived from livers transplanted with Thy1-LSPCs (Fig. 1E). Next, we performed double immunohistochemistry for IL17RB and HNF4α, which revealed that many SHPCs in livers transplanted with Thy1-MCs were strongly stained with the anti-IL17RB antibody; however, the intensity of expression varied between the two clusters (Fig. 2A). Although most HNF4α^+^ hepatocytes, including SHPCs, in livers with or without Thy1-MCs express IL17RB, the level of IL17RB expression was considerably higher in SHPCs transplanted with Thy1-MCs than in other hepatocytes. Additionally, we analyzed the expression of the IL17RB ligand IL17B using immunohistochemistry, which revealed that IL17B expression was observed in SE-1^+^ cells, particularly in SHPC clusters in livers transplanted with Thy1-MCs, but not in those transplanted with Thy1-LSPCs and controls (Fig. 2B). These results indicate that Thy1-MCs can induce SHPC proliferation through IL17RB signaling.

### Characterization of Thy1-MCs and secreted EVs

**Fig. 3:**
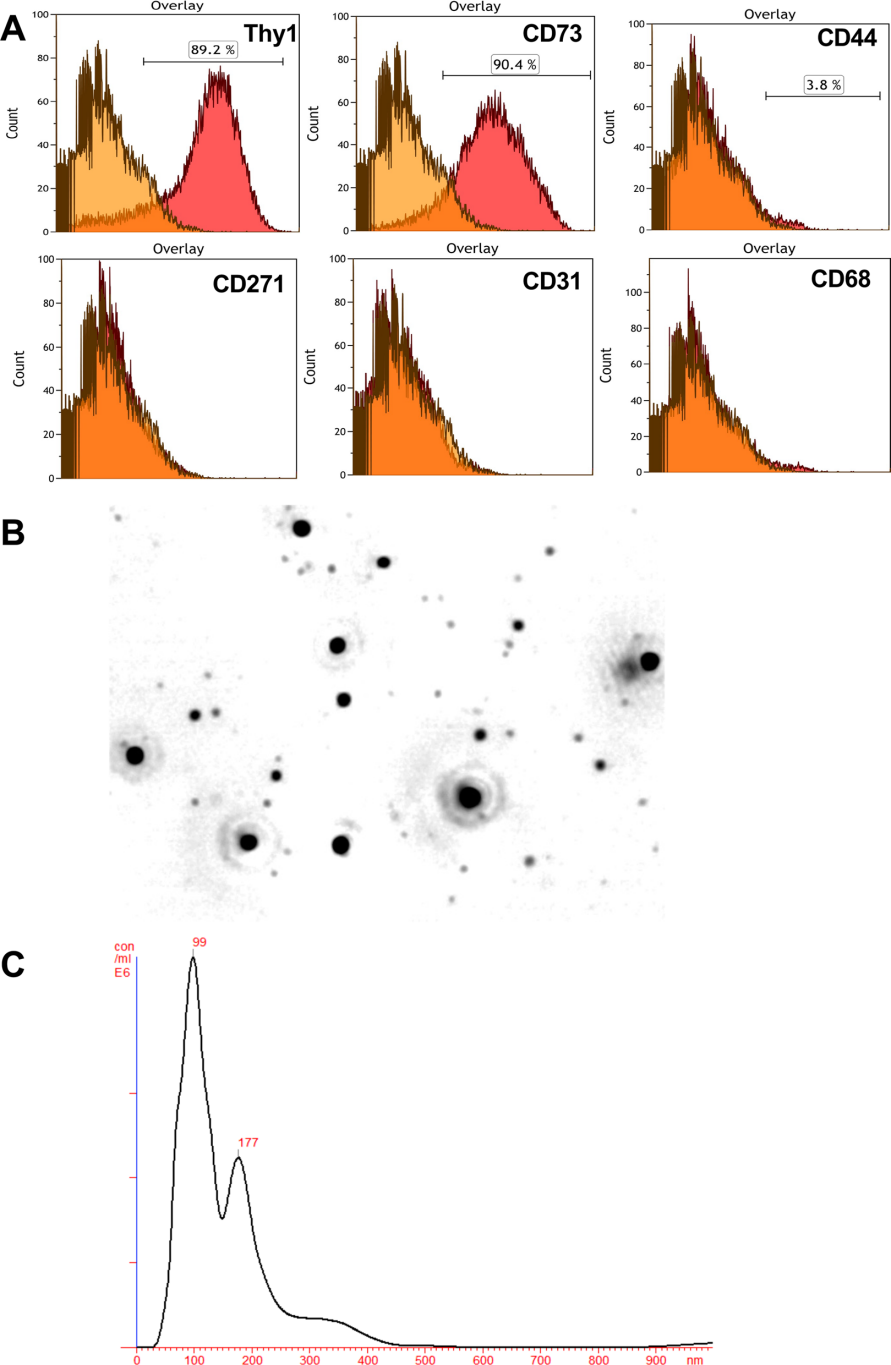
Characterization of Thy1-MCs and EVs. **A** Flow cytometry analysis of cell surface markers (CD90, CD73, CD44, CD31, CD68, and CD271) on BM-MCs. **B** Sample video frame of EVs obtained from cultured Thy1-MCs after 48 h, as analyzed using the NanoSight particle tracking system. **C** Overall size distribution (histograms) and mode (nm)

We evaluated whether Thy1-MCs express mesenchymal stem cell (MSC)-positive markers, such as CD90, CD73, CD44, and CD271 [32], and found that 89.2% and 90.4% of Thy1-MCs expressed CD90 and CD73, respectively, whereas some cells expressed CD44 (3.8%) (Fig. 3A). Thy1-MCs expressed neither CD31^+^ endothelial nor CD68^+^ macrophage cell markers. EVs obtained from the CM of Thy1-MCs cultured for 48 h had an average particle size of ~ 175 nm (Fig. 3B, C). Thy1-MCs (5 × 10^5^ cells) secreted approximately 4.9 × 10^9^ EVs in 48 h, equivalent to 44.0 μg of protein. Proteome analysis revealed that EVs secreted by Thy1-MCs contained CD63, CD81, CD82, CD47, actin, and GAPDH (Additional file 1: Supplemental information) as well as THY1 (CD90) and NT5E (CD73) as membrane proteins of the original cells.

**Fig. 4:**
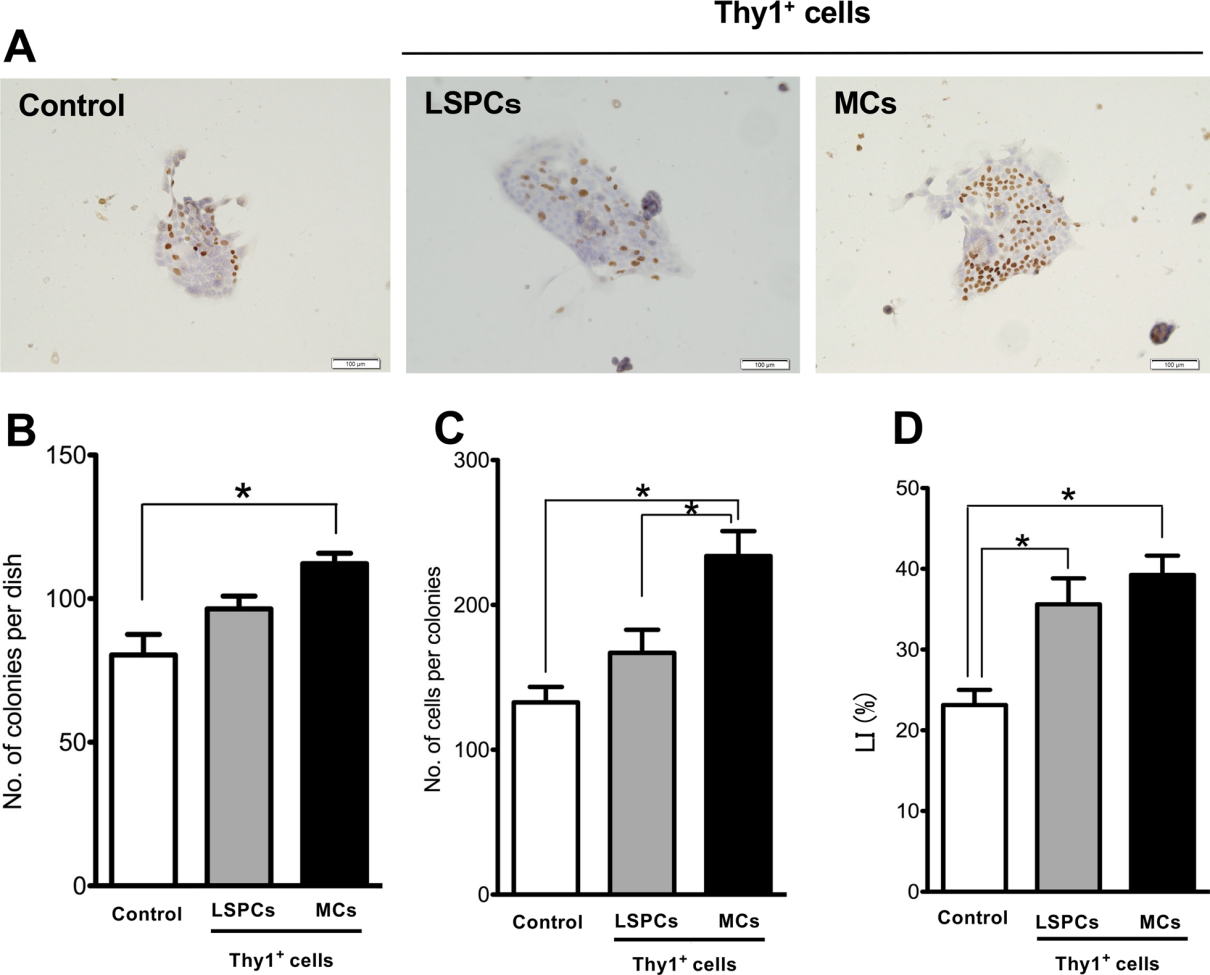
Effect of EVs derived from Thy1-LSPCs and Thy1-MCs. Panel **A** contains photos of typical SH colonies treated with EVs derived from Thy1-LSPCs and Thy1-MCs on day 7 after treatment. Immunocytochemistry for BrdU and hematoxylin staining. Scale bar, 100 μm. **B** Number of SH colonies, **C** number of cells per colony, and  **D** percentage of BrdU^+^ cells per colony. Bars indicate SEs. Asterisks indicate significant differences at *p* < 0.05

Thy1-EVs have been reported to promote SHPC growth [27]. Therefore, we verified whether EVs secreted by Thy1-MCs induce SHPC growth. EVs (1.4 μg) secreted by Thy1-LSPCs (Thy1-LSPC-EVs) and Thy1-MCs (Thy1-MC-EVs) were added to SH culture medium at 3 h after plating, and the medium was replaced with fresh medium without EVs at 48 h after plating. Immunocytochemistry for BrdU demonstrated that the addition of Thy1-MC-EVs accelerated SH growth (Fig. 4A). At 7 days after plating, the number of SH colonies treated with Thy1-MC-EVs was higher than that of colonies treated with Thy1-LSPC-EVs or control EVs (Fig. 4B), and the size of colonies treated with Thy1-MC-EVs was approximately 1.5- and twofold larger than that of colonies treated with Thy1-LSPC-EVs and control EVs, respectively (Fig. 4C). The LI of SH colonies treated with Thy1-MC-EVs was significantly higher than that of SH colonies treated with Thy1-LSPC-EVs and control EVs (Fig. 4D). These results indicate that Thy1-MC-EVs contain factors that can induce the proliferation of HPCs.

### Identification of miRNAs as growth-related agents in EVs secreted by Thy1-MCs

**Fig. 5:**
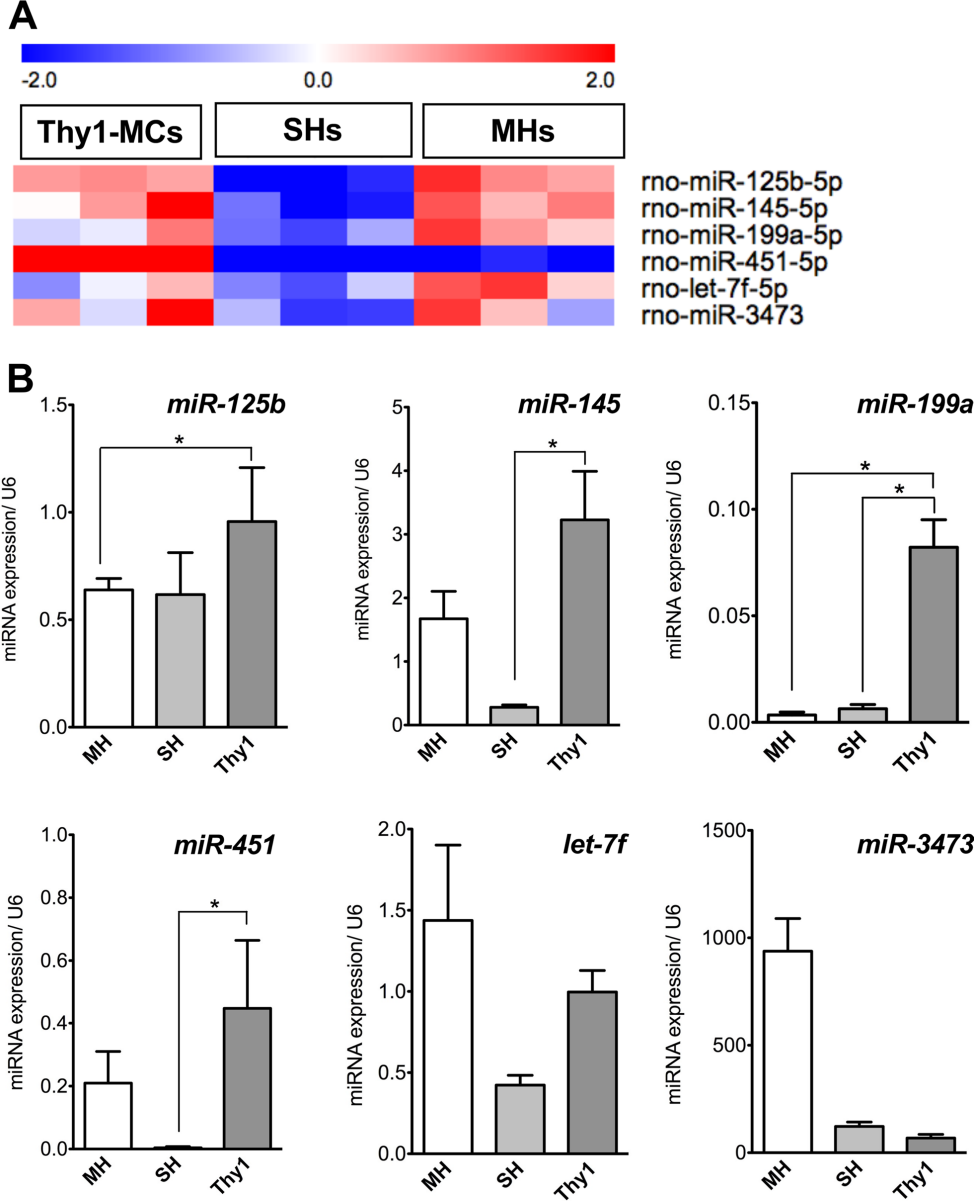
Identification of miRNAs in EVs secreted by Thy1-MCs. **A** Six miRNAs were upregulated by fivefold compared with hepatic Thy1-MCs, as shown in the heatmap. **B** Quantification of *miR-125b-5p*, *miR-145-5p*, *miR-199a-5p*, *miR-451-5p*, *miR-let-7f*, and *miR-3473* expression in EVs secreted by MHs, SHs, and hepatic Thy1-MCs via qRT–PCR. Four of six miRNAs were more abundant in EVs secreted by Thy1-MCs than in other cells. Bars indicate SEs and asterisks indicate significant differences at *p* < 0.05

EVs are known to be enriched with many bioactive molecules, such as proteins, lipids, RNAs, and mitochondrial DNAs [36]. We compared miRNAs in EVs secreted by cultured Thy1-MCs and those secreted by SHs and MHs. We utilized miRNA Oligo Chip to perform a comprehensive analysis of miRNAs and selected miRNAs (*miR-125b*, *miR-145*, *miR-199a*, *miR-451*, *let-7f*, and *miR-3473*) that were expressed at > fivefold higher levels in Thy1-MC-EVs than in SH- and MH-EVs (Fig. 5A). The expression of the selected miRNAs was confirmed via qRT–PCR. The expression of *miR-125b*, *miR-145*, *miR-199a*, and *miR-451* was relatively higher in Thy1-MC-EVs than in SH- and MH-EVs (Fig. 5B). Therefore, these miRNAs were further analyzed.

**Fig. 6:**
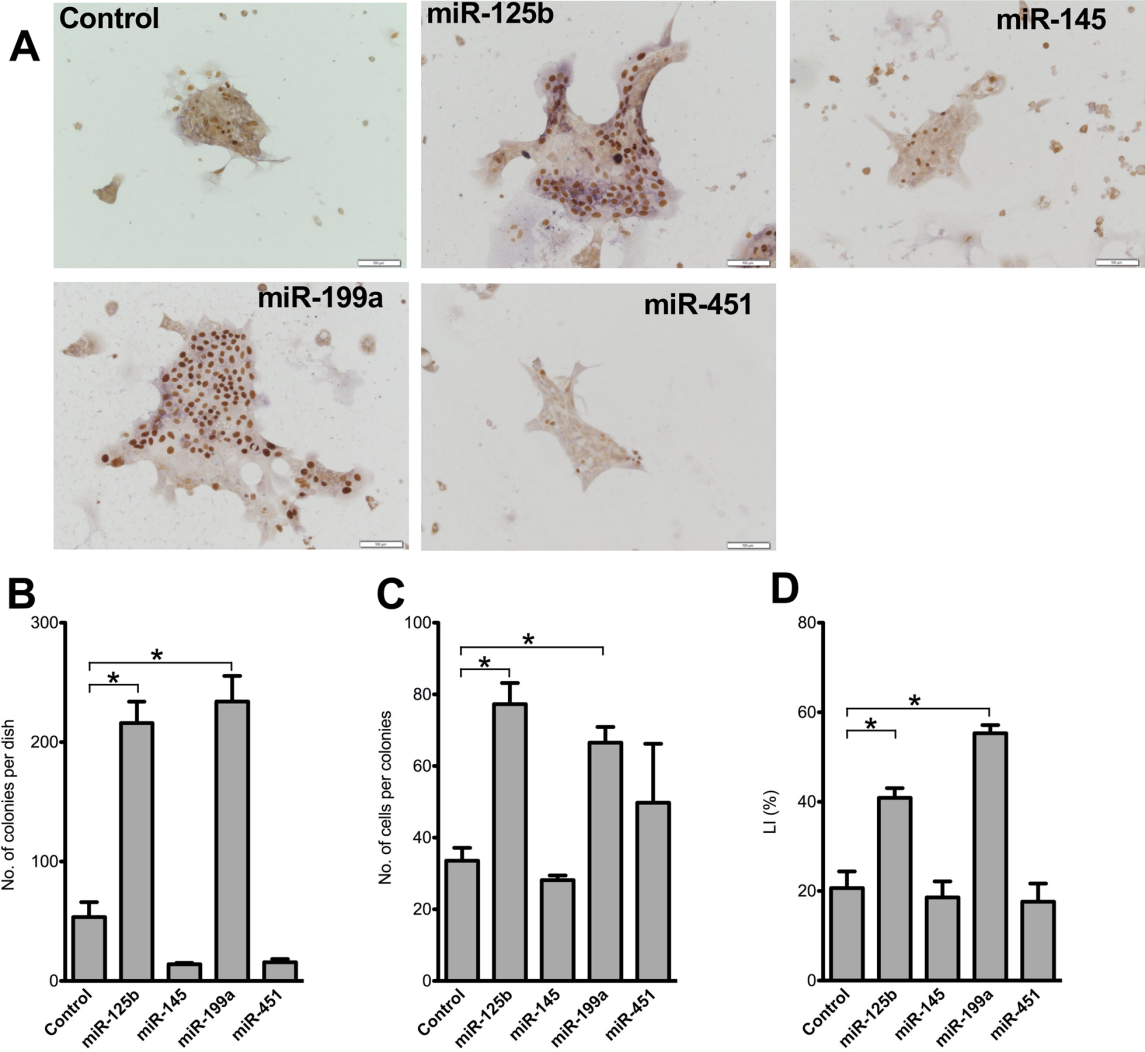
Effect of selected miRNAs in Thy1-MC EVs on SH proliferation. Photos indicate SH colonies transfected with *miR-125b-5p*, *miR-145-5p*, *miR-199a-5p*, and *miR-451-5p* mimics, which were added to the culture medium for 2 days (**A**). The cells were cultured until day 7, and BrdU was added to the culture medium 24 h before fixation. Immunostaining was performed using BrdU. Scale bar, 100 μm. **B** Total number of SH colonies per well, **C** number of cells per colony, and **D** percentage of BrdU^+^ cells per colony (**E**). Asterisk indicates significant differences compared with the control at *p* < 0.05

Mimics of the selected miRNAs were added to cultured SHs, and their effect on SH growth was evaluated (Fig. 6). At 7 days after treatment, the number of colonies per dish and cells per colony significantly increased in cells treated with *miR-125b* and *miR-199a* mimics (Fig. 6A–C). Moreover, the ratio of BrdU^+^ cells significantly increased in cells treated with *miR-125b* and *miR-199a* mimics (Fig. 6D). These results indicate that *miR-125b* and *miR-199a* mimics promote HPC growth. Next, we evaluated whether *miR-125b* and *miR-199a* mimics induce *Il17rb* and *Il17b* expression in SHs and SECs, respectively, and demonstrated that none of them could induce the expression (data not shown).

### Administration of Thy1-MC-EVs transfected with miR-199a-5p to Ret/PH-treated rat livers

**Fig. 7:**
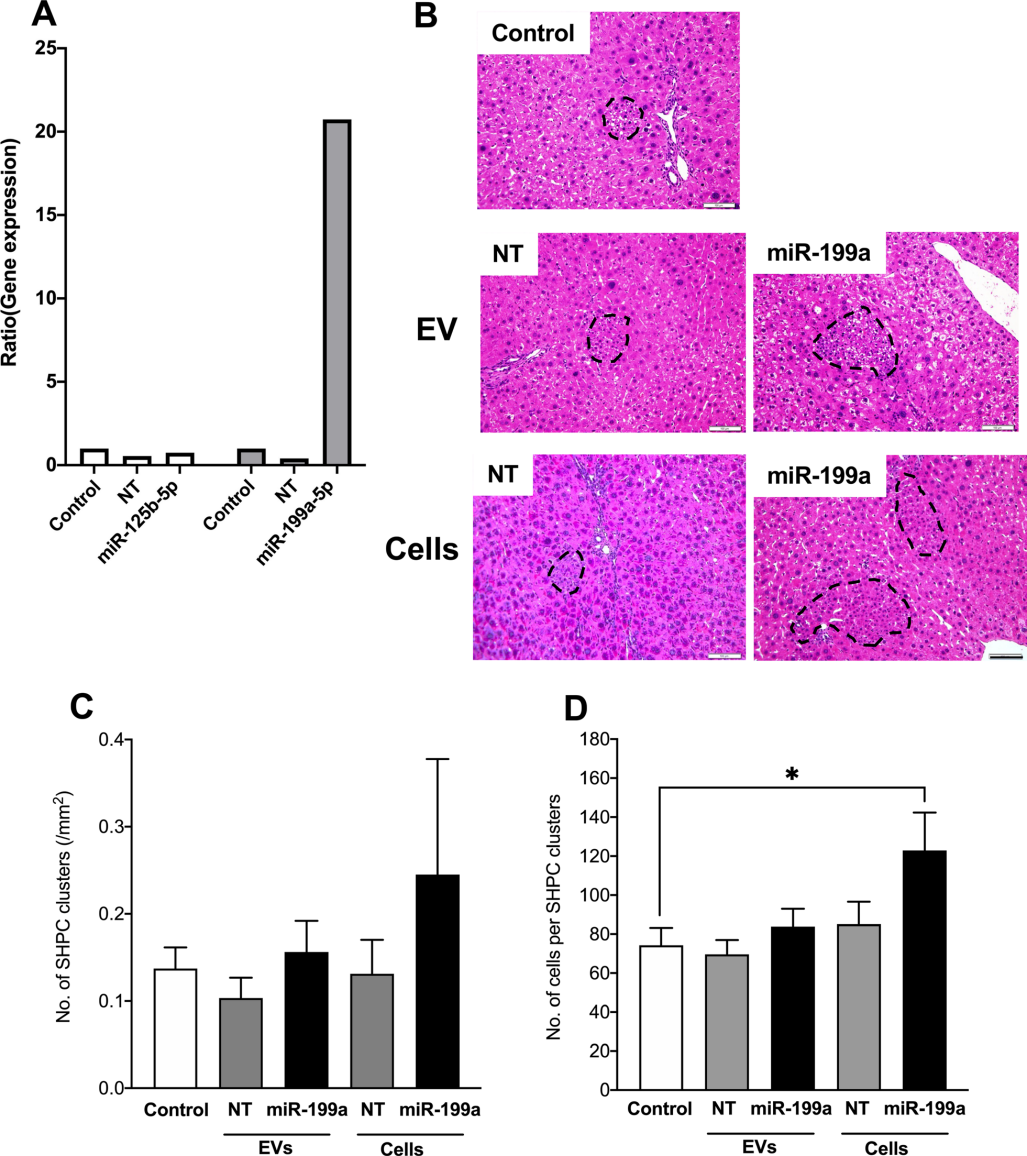
Overexpression of *miR-199a-5p* in Thy1-MC EVs. Administration of Thy1-MC EVs and cells transfected with *miR-199a-5p* to Ret/PH-treated rat livers. EVs obtained from the conditioned medium of cultured Thy1-MCs transfected with *miR-125b-5p* and *miR-199a-5p* for 48 h were administered, and the livers were examined 14 days later. **A** Quantification of *miR125b-5p* and *miR-199a-5p* expression in Thy1-MC EVs with and without transfection via qRT–PCR. Bars indicate SEs. Asterisks indicate significant differences at *p* < 0.05. **B** Photos of SHPC clusters in the livers of control, EVs secreted by Thy1-MCs transfected with negative control (NT), or *miR-199a-5p* (miR-199a). The samples were stained using HE. SHPC clusters are enclosed by dotted lines: scale bar, 200 μm. **C** Number of SHPC clusters in each liver area and **D** number of cells per cluster are shown. Bars indicate SEs and asterisks indicate significant differences at *p* < 0.05

We analyzed the efficiency of *miR-125b* and *miR-199a* overexpression in Thy1-MC-EVs. The level of *miR-199a* expression in EVs secreted by *miR-199a*-transfected Thy1-MCs was ~ 20-fold higher than that in EVs secreted by control and negative target (NT) controls (Fig. 7A**)**. We could not induce *miR-125b* overexpression in Thy1-MCs although several sequences of *miR-125b* mimics were transfected into Thy1-MCs. Thus, we only investigated the effect of *miR-199a*-overexpressing EVs (miR199a-EVs) on SHPC growth. NT-EVs and miR199a-EVs were administered to Ret/PH-treated rat livers through the spleen. miR199a-EVs could neither induce the emergence nor promote the proliferation of SHPCs (Fig. 7B); however, transplantation of *miR-199a*-overexpressing Thy1-MCs could induce SHPC proliferation (Fig. 7B–D).

### Identification of cytokines in EVs secreted by Thy1-MCs

**Fig. 8:**
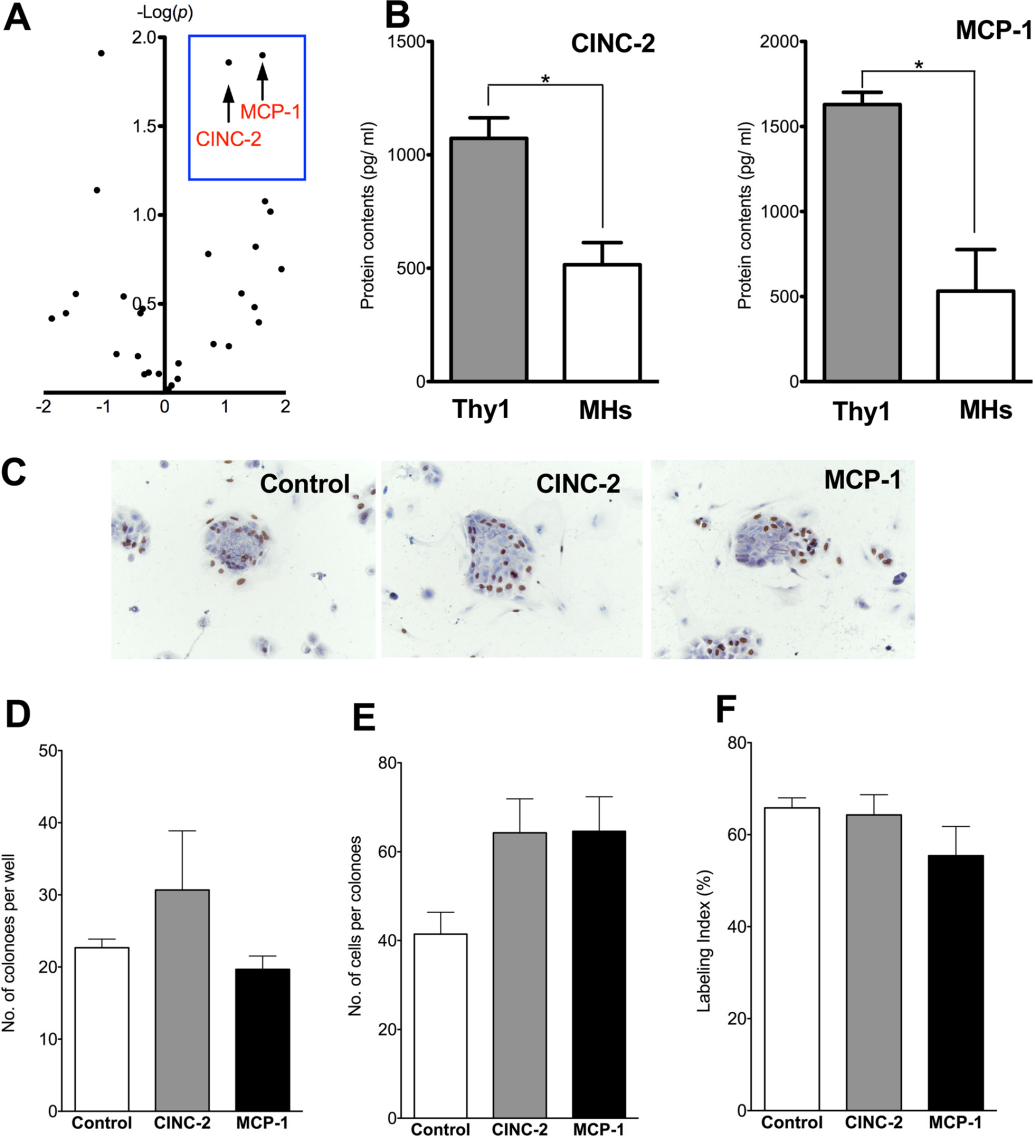
Effects of cytokines in EVs derived from Thy1-MCs. Volcano plot of cytokines given by Thy1-MCs and MHs (**A**). The number of cytokines found in EVs secreted from hepatic Thy1-MCs and MHs was assessed using a custom-designed Quantibody rat-specific protein array (QAR-CAA-67) (**B**). Higher concentrations of CINC-2 and MCP-1 were found in Thy1-MC-EVs than in MH-EVs. CINC-2 and MCP-1 were added to the culture medium 3 h after plating and refreshed every alternate day. Panel **C** demonstrates the photos of typical SH colonies treated with each cytokine. BrdU was added to the culture medium 24 h before fixation, and immunocytochemistry for BrdU was conducted 7 days after plating. Scale bar, 100 μm. The total number of SH colonies per well (**D**) , number of cells per colony (**E**), and percentage of cells with BrdU^+^ nucleus per colony (**F**) are demonstrated. Asterisk indicates significant differences at *p* < 0.05

In addition to miRNAs, Thy1-MC-EVs may contain cytokines that can promote SH growth (Fig. 8). We analyzed the major cytokines secreted by Thy1-MCs using a cytokine array. We selected CINC-2 (Cxcl3) and MCP-1 (Ccl2) because they were expressed at twofold higher levels in Thy1-MCs than in MHs (Fig. 8A). We then measured the levels of cytokines secreted by Thy1-MC-EVs and MH-EVs (Fig. 8B). The levels of CINC-2 and MCP-1 were considerably higher in Thy1-MC-EVs than in MH-EVs. Further, we treated SHs with CINC-2 and MCP-1 individually and found that none of them could promote SH growth (Fig. 8C–F).

**Fig. 9:**
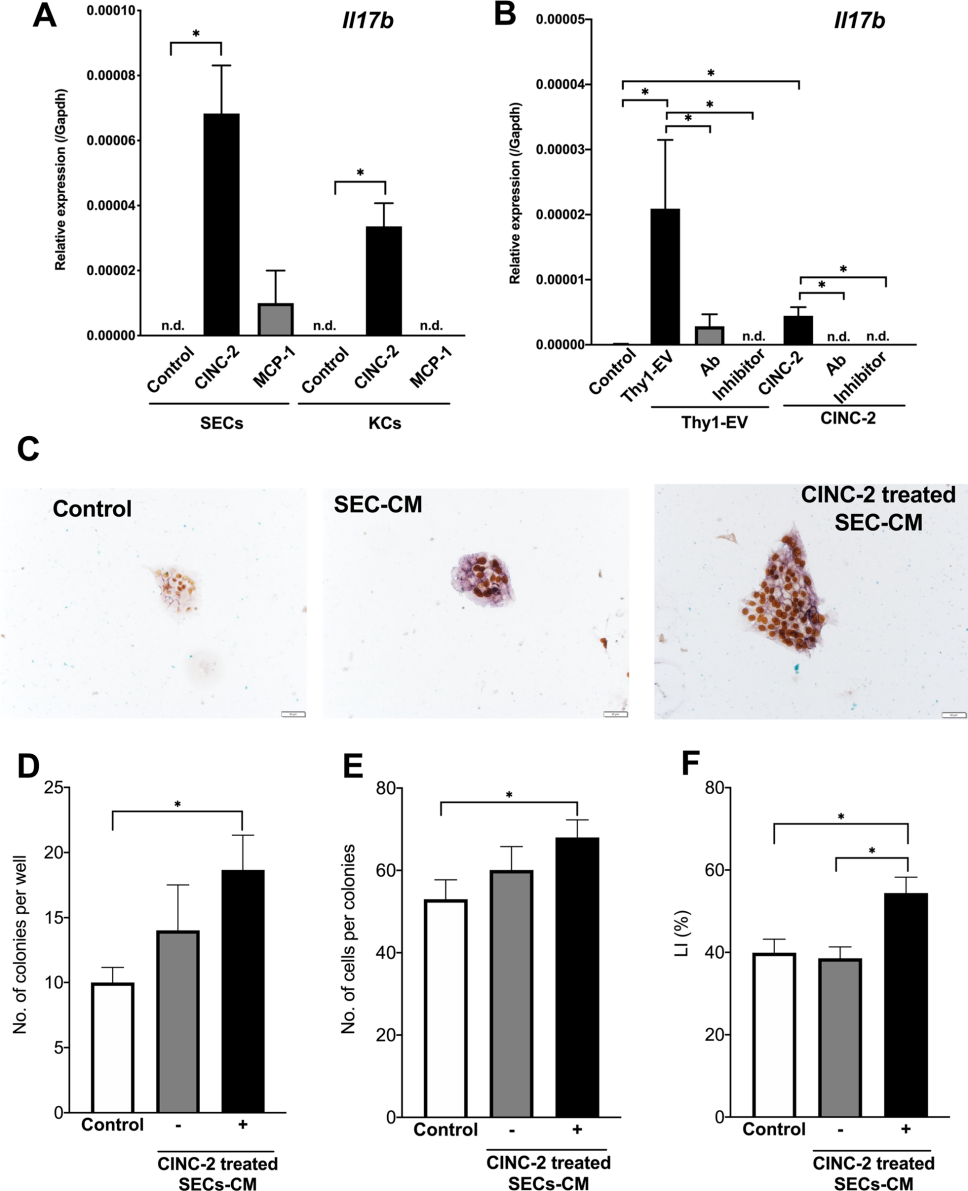
Induction of *Il17b* expression in SECs by cytokines in Thy1-MC EVs. Administration of CINC-2 and MCP-1 to SECs and Kupffer cells (**A**). EVs were prepared from the conditioned medium of Thy1-MCs cultured for 2 days and were administered to SECs and Kupffer cells isolated from a normal rat liver. Thy1-EVs were administered 3 h after plating, and the cells were cultured for 48 h. Using a CINC-2 neutralizing antibody or CXCR2 inhibitor (SB225001), the effects of CINC-2/CXCR2 signaling were assessed for the induction of *Il17b* expression in SECs by CINC-2 and Thy1-EVs, respectively. *Il17b* expression was measured via quantitative reverse transcription polymerase chain reaction (qRT–PCR) (**B**). SHs were cultured in serum-free media on Matrigel-coated dishes. SEC-CM with and without CINC-2 treatment were administered to SHs for 3 h after plating and cultured for 7 days. *Il17rb* expression was measured via qRT–PCR (**C**). Asterisk indicates significant differences at *p* < 0.05. *EVs* extracellular vesicles, *MHs* mature hepatocytes, *SECs* sinusoidal endothelial cells, *SHs* small hepatocytes. Panel **C** contains the photos of typical SH colonies treated with each cytokine. BrdU was added to the culture medium 24 h before fixation, and immunocytochemistry for BrdU was performed 7 days after plating. Scale bar, 100 μm. Total number of SH colonies per well (**D**), number of cells per colony (**E**), and percentage of cells with BrdU^+^ nucleus per colony (**F**). Asterisk indicates significant differences at *p* < 0.05

Next, we determined whether these cytokines can induce *Il17b* expression in SECs and KCs. CINC-2 could induce *Il17b* expression in SECs and KCs, whereas MCP-1 could not (Fig. 9A). To verify whether *Il17b* expression was induced by CINC-2/CXCR2 signaling, we added neutralizing antibodies against CINC-2 or CXCR2 inhibitor (SB225001) to SECs treated with Thy1-EVs or CINC-2 (20 ng/mL). Thy1-EVs induced *Il17b* expression in SECs (Fig. 9B), which was suppressed by the neutralizing antibody or SB225001. Although CINC-2 also induced *Il17b* expression in SECs, its extent of induction was considerably lower than that of Thy1-EVs. The neutralizing antibodies against CINC-2 and SB225001 significantly inhibited *Il17b* expression in SECs. These results indicate that CINC-2 in Thy1-EVs plays a major role in inducing IL17B production in SECs through CINC-2/CXCR2 signaling.

Furthermore, we determined whether SECs treated with CINC-2 (CINC-2-SEC) can promote HPC growth. SEC-CM and CINC2-SEC-CM were added to SH culture medium. CINC-2-SEC-CM was found to accelerate SH growth (Fig. 9C). At 7 days after plating, the number of SH colonies in treated with CINC-2-SEC-CM increased approximately twofold compared with that in the control (Fig. 9D). Moreover, the size of the colonies in treated medium was significantly larger than that in the control (Fig. 9E). Additionally, SHs treated with CINC-2-SEC-CM showed higher LI than the control (Fig. 9F). Considering that IL17B administration accelerated SH growth, these results indicate that CINC-2 in Thy1-EVs induces SECs to secrete IL17B, thereby accelerating HPC growth.

### Activation of KCs by Thy1-EVs

**Fig. 10:**
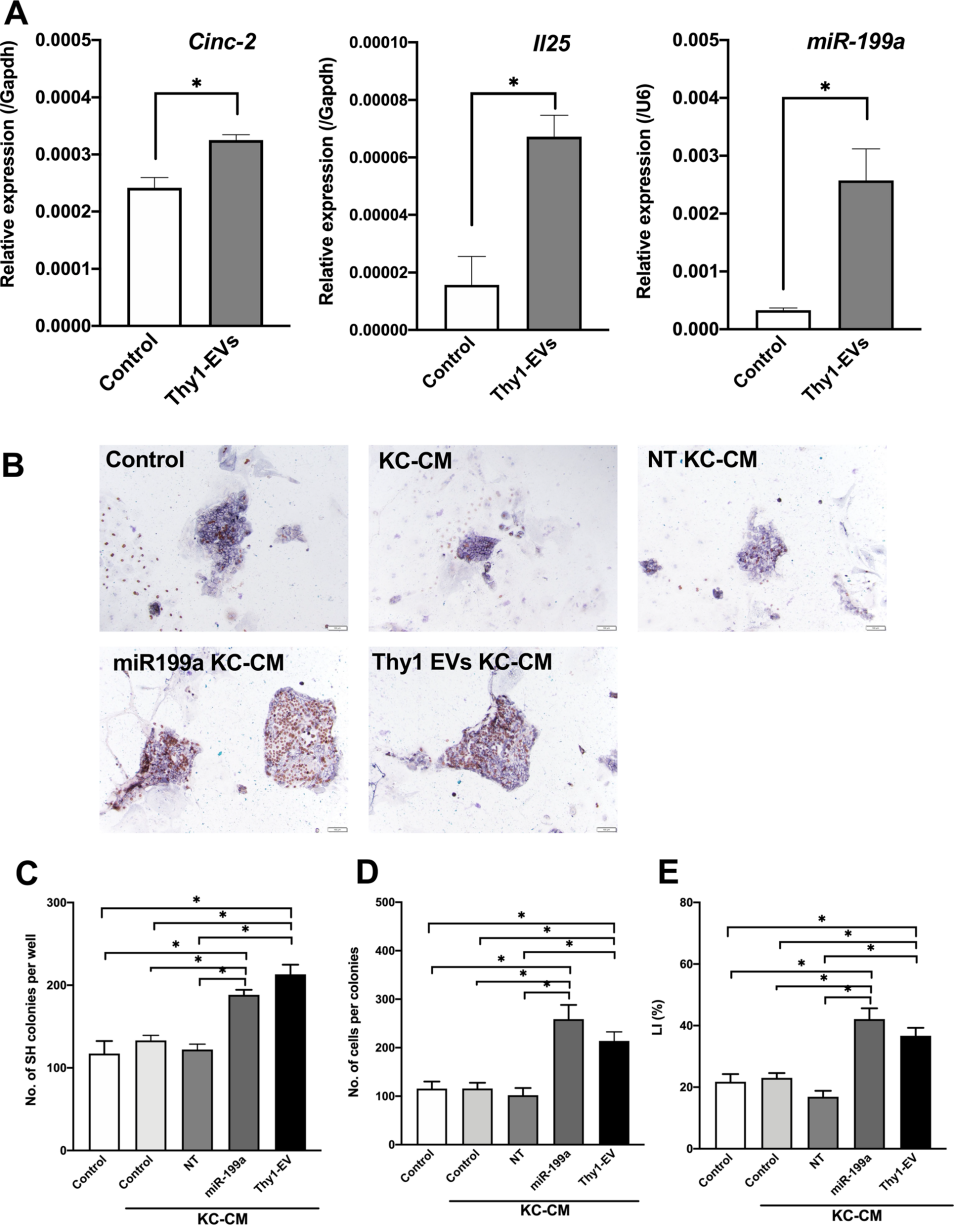
Activation of KCs by Thy1-EVs. Thy1-EVs were administered for 3 h after plating, and the cells were cultured for 48 h. Expression of *Cinc-2*,* Il25*, and *miR-199a-5p* was measured using quantitative reverse transcription polymerase chain reaction. Asterisk indicates significant differences at *p* < 0.05 (**A**). SHs were cultured in serum-free media on Matrigel-coated dishes. KC-CM with and without *miR-199a-5p* mimics and Thy1-EVs were added to SHs 3 h after plating and cultured for 7 days. *CM* conditioned medium, *EV* extracellular vesicle, *KC* Kupffer cell, *SHs* small hepatocytes. Panel **B** contains the photos of typical SH colonies treated with each CM. BrdU was added to the culture medium 24 h before fixation, and immunocytochemistry for BrdU was performed 7 days after plating. Scale bar, 100 μm. Total number of SH colonies per well (**C**), number of cells per colony (**D**), and percentage of cells with BrdU^+^ nucleus per colony (**E**) are shown. Asterisk indicates significant differences at *p* < 0.05

Consistent with a previous study [27], the current study demonstrated that IL17B, IL25, CINC-2, *miR-125b*, and *miR-199a* play important roles in HPC growth. Therefore, we examined whether Thy1-EVs can increase the expression of these inducers in KCs and SECs. In cultured KCs, Thy1-EVs upregulated the expression of CINC-2, IL25, and *miR-199a* compared with control EVs (Fig. 10A). Although CINC-2 could induce *Il17b* expression in KCs, *IL17b* expression was not induced by Thy1-EVs (Fig. 9A).

Next, we determined whether KCs treated with *miR-199a* mimics and Thy1-EVs can promote HPC growth. KC-CM, NT-CM, miR-199a-KC-CM, and Thy1-EVs-KC-CM were added to SH culture medium. miR-199a-KC-CM and Thy1-EVs-KC-CM were found to accelerate SH growth (Fig. 10B). At 7 days after plating, the number of SH colonies in treated medium increased approximately 1.5-fold compared with that in the control (Fig. 10C), and the size of the colonies in treated medium was significantly larger than that in the control (Fig. 10D). Additionally, SHs treated with miR-199a-KC-CM and Thy1-EVs-KC-CM showed higher LI than the control (Fig. 10E). Considering that the administration of *miR-199a* mimics accelerated SH growth (Fig. 6), these results indicate that Thy1-EVs induce KCs to secrete IL25 and *miR199a*, thereby accelerating HPC growth.

## Discussion

We previously reported that Thy1^+^ cells isolated from GalN-treated livers contain a heterogeneous population primarily consisting of MCs and LSPCs [20] and that the transplantation of Thy1^+^ cells and administration of Thy1-EVs activate the proliferation of SHPCs in Ret/PH-treated livers [27]. In the current study, we clarified that Thy1-MCs-secreted EVs can promote the expansion of SHPC clusters. As the specific cell surface markers of rat hepatic stellate cells (HSCs) have not yet been identified, we could not perform an independent experiment using sorted cells. However, consistent with a previous study [32], desmin^+^ cells in livers were not immunohistochemically stained with IL17B or IL25. In addition, SE-1^−^/CD68^−^ cells, which were sorted from nonparenchymal cells and included HSCs, did not express *Il17b* or *Il25* when Thy1-EVs were added to the culture medium. Therefore, we focused on the roles of both SECs and KCs in the growth regulation of SHPCs in this study. Furthermore, the inactivation of KCs by Gadolinium chloride (GdCl_3_) does not affect the growth of SHPCs in livers transplanted with Thy1^+^ cells [32], indicating that the phagocytic function of KCs plays an important role in the expansion of SHPCs. However, factors in Thy1-EVs that are crucial for activating IL17RB signaling and the mechanism by which they induce SHPC expansion remain unclear. In the current study, we first demonstrated that EVs secreted by Thy1-MCs, not by Thy1-LSPCs, play an important role in the acceleration of liver regeneration. Second, we determined that CINC-2 contained in Thy1-MC-EVs upregulated the production of IL17B in SECs and that KCs stimulated by Thy1-MCs could secrete CINC-2. Third, similarly to CINC-2, we determined that *miR-199a-5p* contained in Thy1-MC-EVs directly stimulate the proliferation of SHPCs and that KCs stimulated by Thy1-MCs could secrete *miR-199a-5p*.

CINC-1, CINC-2, and CINC-3 belong to the CXC chemokine family and are potent chemotactic factors for neutrophils [37, 38]. CINCs recruit neutrophils through CXCR2 receptor signaling [39]. In this study, we revealed that CINC-2 can induce *Il17b* expression in SECs and KCs; however, the induction was suppressed by neutralizing antibodies against CINC-2 and SB225001. These findings indicate that IL17B production in SECs and KCs is regulated by CINC-2/CXCR2 signaling. It is well known that lung-resident MSCs (LR-MSCs) contain a population of cells that can differentiate into alveolar epithelial type II cells [40]. Similar to bone marrow-derived MSCs, LR-MSCs express Thy1 in addition to Sca-1, CD29, CD44, and CD106. Rat alveolar epithelial type II cells reportedly secrete CINC-2 and MCP-1 in response to a combination of IL-1β, TNF-α, and IFN-γ [41]. Hence, it is possible that EVs secreted by Thy1^+^ LR-MSCs contain CINC-2 and MCP-1. We revealed that Thy1-MCs, which localize within the Glisson’s sheath in healthy rat livers, emerged in the lobules after GalN treatment and secreted EVs containing CINC-2 and MCP-1. Although portal Thy1^+^ cells isolated from healthy livers did not express CINC-2, TNF-α could induce the expression of CINC-2 in Thy1^+^ cells (data not shown). These results suggest that Thy1-MCs can secrete CINC-2 due to an inflammatory response. In this study, we showed that CINC-2 could act as a factor for inducing IL17B as well as a chemotactic factor for neutrophils.

Although CINC-2 cannot directly induce SH growth, the CM of CINC-2-treated SECs can promote SH proliferation. Additionally, SH growth was inhibited by the administration of a neutralizing antibody against IL17B and CXCR2 inhibitor. In livers transplanted with Thy1-MCs, SHPCs were stained intensively for IL17RB, and SECs in the clusters distinctly expressed IL17B. These findings indicate that SECs secrete IL17B via the CINC-2/CXCR signaling pathway and that the secreted IL17B promotes the growth of IL17RB^+^ SHPCs. In this study, EVs secreted by Thy1-MCs contained MCP-1 as well as CINC-2. Although we attempted to clarify the role of MCP-1 in the growth of HPCs, we observed that MCP-1 was neither involved in IL17RB signal transduction nor in the direct effect of HPC proliferation. It was reported that MCP-1 expression was highly upregulated by thioacetamide in mouse models of acute liver failure [42]. Transplantation of CCR2-overexpressed MSCs revealed that the donor cells rapidly migrated and localized to the injured areas and remained in the liver lesions for a longer time than the control cells, which promoted liver regeneration by the alleviation of liver injury with reduced inflammatory infiltration and hepatic apoptosis. Further, Chen et al. [43] demonstrated that human menstrual blood-derived stem cells can induce MCP-1 expression in HSC coculture, which suppressed the proliferation of activated HSCs. Although the relationship between MCP-1 and HSCs may play some roles in the current study, we did not perform any experiment to verify them. Hence, further experiments are warranted to verify the involvement of MCP-1 in the regeneration of livers transplanted with Thy1-MCs.

EVs are enriched with many bioactive molecules, such as proteins, lipids, RNA, and mitochondrial DNA [36]. Previously, we discovered that *miR-146a-5p* in EVs secreted by BM-MCs plays an important role in SHPC activation [32]. Therefore, we initially hypothesized that miRNAs in EVs secreted by Thy1-MCs are involved in SHPC growth. We selected *miR-125b*, *miR-145*, *miR-199a*, *and miR-451* based on the results of miRNA microarray and qRT–PCR analysis and identified *miR-125b* and *miR-199a* as factors that can enhance HPC proliferation by treating SHs with their mimics. It has been reported that *miR-125b* and *miR-199a* are associated with the growth regulation of hepatocytes [44–47]. However, the results regarding the effects of both miRNAs were inconsistent. Although Liu et al. [44] reported that *miR-199a* negatively regulated the growth of hepatocellular carcinoma cells by targeting CDC25A, *miR-199a* positively regulated hepatocyte proliferation by targeting TNF-α/TRADD/caspase 3 signaling, resulting in the acceleration of rat liver regeneration following 70%PH [45]. Contrarily, Hua et al. [46] reported that *miR-125b* suppressed the proliferation of hepatocellular carcinoma cells by targeting TXNRD1, whereas Yang et al. [47] showed that *miR-125b* overexpression by adeno-associated virus promoted the proliferation of mouse hepatocytes following 70% PH. However, in the current study, although we attempted to overexpress *miR-125b* in Thy1-MCs, commercial *miR-125b* vectors could not enhance the production of *miR-125b* in cells. It remains unclear whether *miR-125b* is involved in IL17RB signaling. Additionally, it is necessary to clarify the reason for the difficulty of inducing *miR-125b* overexpression in Thy1-MCs.

In this study, we revealed that *miR-199a* is involved in the regulation of HPC growth. Although the administration of *miR-199a* mimics promoted the growth of cultured SHs, *Il17rb* and *Il17b* were not expressed in SHs. Moreover, when miR199a-EVs secreted by *miR-199a*-overexpressing Thy1-MCs were administered to Ret/PH-treated rat livers, they could neither induce the emergence nor promote the expansion of SHPC clusters. Contrarily, the transplantation of *miR-199a*-overexpressing Thy1-MCs into Ret/PH-treated rat livers significantly promoted the expansion of SHPCs. Additionally, KCs treated with Thy1-EVs can upregulate the expression of *Cinc-2*, *Il25*, and *miR-199a-5p*. Moreover, when the cultured KCs were treated with either *miR-199a* or Thy1-EVs, both CMs could improve SH growth. However, when GdCl_3_ was administered to Ret/PH-treated rats before Thy1^+^ cell transplantation, the effect of growth stimulation on SHPCs was nullified [32]. Considering that KC activation caused by Thy1^+^ cell transplantation is important for the growth of SHPCs, a single administration of Thy1-EVs (i.e., transient exposure to *miR-199a-5p*) to SHPCs may be insufficient to promote in vivo SHPC growth. Further, Nguyen et al. [48] reported that KCs can regulate liver regeneration through CXCR2 signaling in acute liver disease. Although we did not examine the involvement of *miR-199a* in cell growth, Chen et al. [49] reported that *miR-199a* expression was associated with the differentiation of monocytes and macrophages. Thus, we hypothesized that phagocytosis of Thy1^+^ cells by KCs promotes the expansion of SHPC clusters. Considering these results, phagocytosis of Thy1-MCs by KCs may play a crucial role in the regulation of SHPC growth.

## Conclusions

**Fig. 11:**
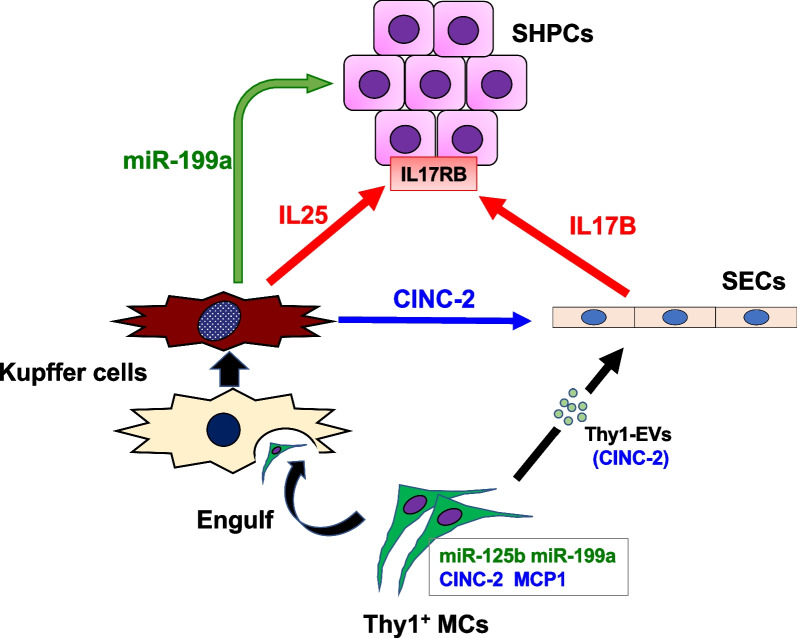
Mechanism of promoting SHPC growth by the transplantation of Thy1-MCs. IL17B, IL25, CINC-2, *miR-125b*, and *miR-199a* are identified as growth inducers of HPCs in liver regeneration induced by the transplantation of Thy1-MCs. The interactions between SHPCs, SECs, and KCs and the inducers are summarized as follows: (1) transplanted Thy1-MCs transiently remain in sinusoids and secrete EVs containing *miR-199a-5p* and CINC-2. (2) EVs directly affect SHPCs, resulting in the induction of IL17RB expression and promotion of SHPC growth by miRNAs in Thy1-MC-EVs. (3) EVs directly affect SECs, resulting in IL17B production via the CINC-2/CXCR2 signaling pathway. (4) KCs engulf Thy1-MCs to become activated, resulting in the secretion of EVs containing *miR-199a-5p* and CINC-2 in addition to IL25. (5) CINC-2 and *miR-199a-5p* in EVs secreted by KCs induce IL17B production in SECs and promote SHPC growth, respectively. (6) Promotion of SHPC growth via SECs and KCs persists for at least 2 weeks after transplantation of Thy1-MCs

In this study, we indicated that Thy1-MC transplantation accelerates liver regeneration due to the expansion of SHPC clusters, which is stimulated by CINC-2/IL17RB signaling and *miR-199a-5p* via the activation of both SECs and KCs, and the inducers are summarized as Fig. 11. These findings may provide valuable evidence to facilitate the development of therapeutics based on the substances produced by engineered cells for severe liver diseases, rather than the transplantation of these cells. Further experiments should be performed to elucidate the roles of *miR-125b* and MCP-1 and detailed mechanisms of *miR-199a* and IL17RB signaling in liver regeneration by Thy1-MC transplantation.

## Supplementary Information

**Additional file 1.** Proteomic data of Thy1-EVs.

**Additional file 2.** List of antibodies and primers used in this experiment.

## Data Availability

All miRNA microarray data analyzed during the current study are registered in the GEO database (Accession No. GSE222517). The mass spectrometry proteomics data are available in the Proteome Xchange Consortium database (Accession No: PXD039384).

## Abbreviations

LSPCs: Liver stem/progenitor cells
GalN: D-galactosamine
PH: Partial hepatectomy
SHs: Small hepatocytes
MHs: Mature hepatocytes
MCs: Mesenchymal cells
SHPCs: Small hepatocyte-like progenitor cells
Ret: Retrorsine
IL: Interleukin
RB: Receptor B
SECs: Sinusoidal endothelial cells
KCs: Kupffer cells
EVs: Extracellular vesicles
CINC-2: Cytokine-induced neutrophil chemoattractant-2
MCP-1: Monocyte chemotactic protein 1
DPPIV: Dipeptidyl peptidase IV
CM: Conditioned medium
HPCs: Hepatocytic progenitor cells
