# Supplementary material for: CINC-2 and miR-199a-5p in EVs secreted by transplanted Thy1+ cells activate hepatocytic progenitor cell growth in rat liver regeneration
Source: Stem Cell Res Ther. 2023 May 16;14:134. doi: 10.1186/s13287-023-03346-z (PMC10190025; doi:10.1186/s13287-023-03346-z)
Supplement: Supplementary file 2 — Additional file 2. List of antibodies and primers used in this experiment. [file 13287_2023_3346_MOESM2_ESM.docx]

**Table S1** List of antibodies used in the experiments.

| Antibodies | Host | Supplier | Dilution |
| --- | --- | --- | --- |
| CD90 (Thy1) | Mouse | Serotec, Raleigh, NC (cat. no. MCA47R) | 1:500 |
| CD73 | Mouse | BD Biosciences Pharmingen, Franklin Lakes, NJ (cat. no. 551123) | 1:200 |
| CD44 | Mouse | BD Biosciences Pharmingen, Franklin Lakes, NJ (cat. no. 554869) | 1:1000 |
| CD68 | Mouse | Serotec, Raleigh, NC (cat. no. MCA341R) | 1:500 |
| CD271 | Mouse | Abcam, Cambridge, UK (cat.no. ab6172) | 1:100 |
| CD31 | Mouse | Abcam, Cambridge, UK (cat.no. ab64543) | 1:100 |
| IL17RB | Rabbit | Bioss, Boston, USA (cat. no. bs-2610R) | 1:500 |
| IL17B | Rabbit | Cloud-Clone Corp, USA (cat. no. PAB700Ra01) | 1:200 |
| Hepatocyte nuclear factor 4α (HNF4α) | Goat | Santa Cruz, Santa Cruz, CA (cat. No. sc-6556) | 1:200 |
| SE-1 | Mouse | Immuno-Biological Lab., Takasaki, Japan (cat. no. 10078) | 1:200 |
| BrdU | Mouse | DakoCytomation, Glostrup, Denmark (cat. no. M0744) | 1:200 |
| Mouse IgG2a+b microbeads | Rat | Miltenyi Biotec, Bergisch Gladbach, Germany (cat. no. 130-047-201) | 1:5 |
| Alexa 488-conjugated anti-mouse | Goat | Molecular Probes, Eugene, OR (cat. no. A11029) | 1:500 |
| Alexa 488-conjugated anti-mouse | Rabbit | Molecular Probes, Eugene, OR (cat. no. A11059) | 1:500 |
| Alexa 594-conjugated anti-mouse | Goat | Molecular Probes, Eugene, OR (cat. no. A11005) | 1:500 |
| Alexa 488-conjugated anti-rabbit | Donkey | Molecular Probes, Eugene, OR (cat. no. A21206) | 1:500 |
| Alexa 594-conjugated anti-rabbit | Donkey | Molecular Probes, Eugene, OR (cat. no. A21207) | 1:500 |
| Alexa 594-conjugated anti-goat | Donkey | Molecular Probes, Eugene, OR (cat. no. A11058) | 1:500 |
| Biotin-conjugated anti-mouse | Horse | Vector Laboratories, Burlingame, CA (cat. no. BA-2000) | 1:200 |

**Table S2** List of primers used in the experiments of real-time PCR.

| Genes | Real-Time PCR Primers(Assay ID) |
| --- | --- |
| IL17rb | Rn01450514_m1 |
| IL17b | Rn01501362_m1 |
| IL25 | Rn04244818_m1 |
| Cinc-2(Cxcl3) | Rn01414231_m1 |
| Gapdh | Rn01775763_g1 |
| miR-125b-5p | 000449 |
| miR-145-5p | 002278 |
| miR-199a-5p | 000498 |
| miR-451-5p | 001141 |
| let-7f-5p | 000382 |
| miR-3473 | 475642 |
| U6 | 001973 |
